# Discovery and Reconstitution of the Cycloclavine Biosynthetic Pathway—Enzymatic Formation of a Cyclopropyl Group**

**DOI:** 10.1002/anie.201410002

**Published:** 2015-02-25

**Authors:** Dorota Jakubczyk, Lorenzo Caputi, Anaëlle Hatsch, Curt A F Nielsen, Melanie Diefenbacher, Jens Klein, Andrea Molt, Hartwig Schröder, Johnathan Z Cheng, Michael Naesby, Sarah E O'Connor

**Affiliations:** Department of Biological Chemistry, John Innes CentreColney Lane, Norwich (UK); Evolva SADuggingerstrasse 23, Reinach (Switzerland); BASF SEGVF/D—A030, Ludwigshafen (Germany)

**Keywords:** biosynthesis, cyclopropyl group, ergot alkaloids, natural products, pathway reconstitution

## Abstract

The ergot alkaloids, a class of fungal-derived natural products with important biological activities, are derived from a common intermediate, chanoclavine-I, which is elaborated into a set of diverse structures. Herein we report the discovery of the biosynthetic pathway of cycloclavine, a complex ergot alkaloid containing a cyclopropyl moiety. We used a yeast-based expression platform along with in vitro biochemical experiments to identify the enzyme that catalyzes a rearrangement of the chanoclavine-I intermediate to form a cyclopropyl moiety. The resulting compound, cycloclavine, was produced in yeast at titers of >500 mg L^−1^, thus demonstrating the feasibility of the heterologous expression of these complex alkaloids.

The ergot alkaloids, produced by filamentous fungi, are an important class of indole alkaloids with a range of pharmacological and agrochemical activities.[1,2] All ergot alkaloids are derived from the common biosynthetic intermediate chanoclavine-I (**2**), and the structural diversity within the ergot alkaloids results from the elaborate chemical derivatization of this intermediate.[2] However, the mechanisms of most of these downstream elaborations are unknown. Notably, the biosynthetic pathway of cycloclavine (**6**), which contains an unusual cyclopropyl moiety (Figure 1 a), remains cryptic.[3] Herein we report the discovery of the biosynthetic pathway of cycloclavine (**6**) and the reconstitution of this eight-enzyme pathway in *Saccharomyces cerevisiae* at excellent production levels (>500 mg L^−1^). We further propose possibilities for the mechanistic basis of cyclopropyl formation in cycloclavine biosynthesis by the analysis of three enzymes in vitro.

**Figure 1:**
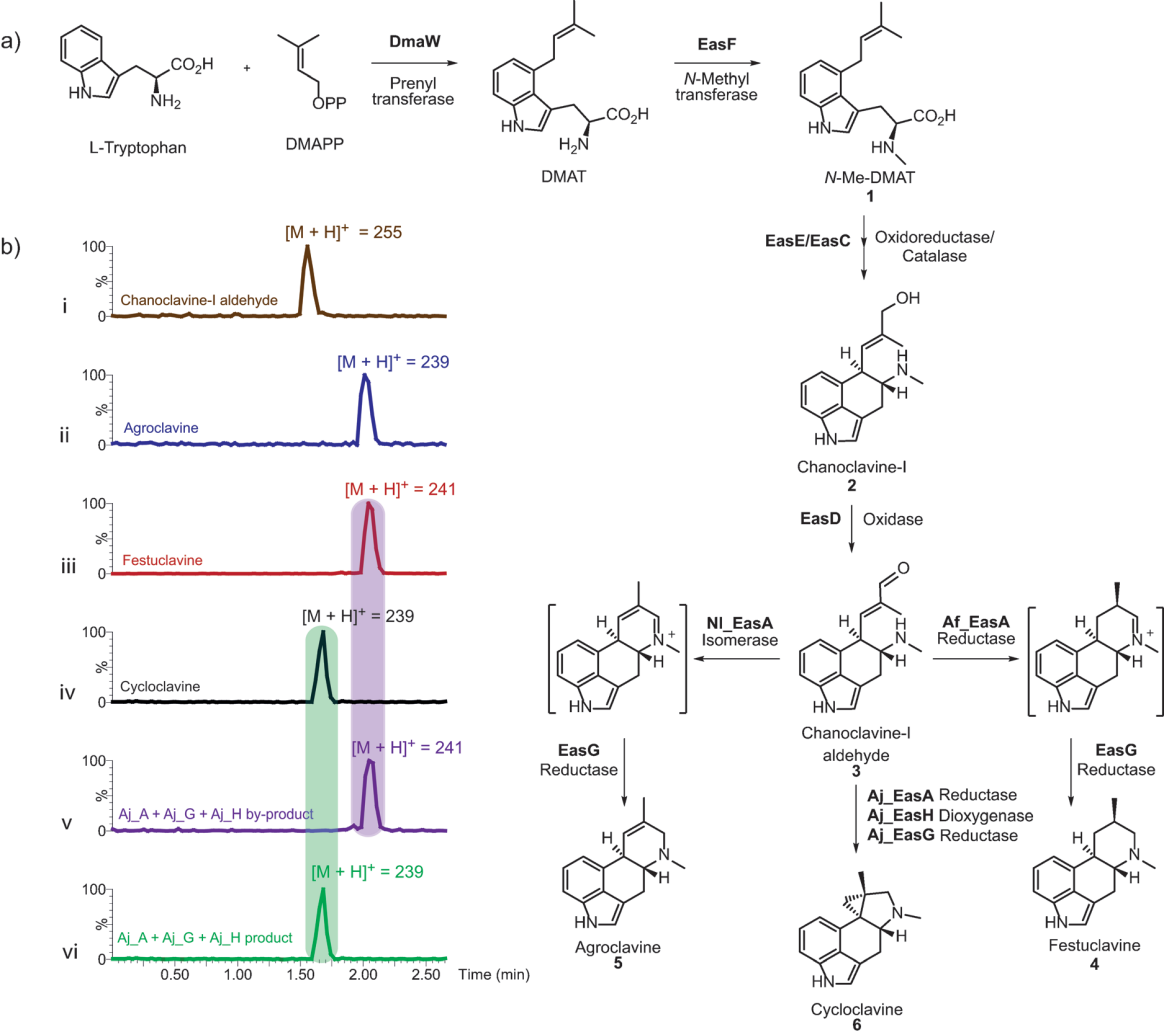
Ergot-alkaloid biosynthetic pathway. a) Biosynthesis of festuclavine (4), agroclavine (5), and cycloclavine (6) from l-tryptophan and dimethylallyl pyrophosphate (DMAPP). b) LC–MS chromatograms showing that EasA, EasG, and EasH are required to generate cycloclavine (6) from chanoclavine-I aldehyde (3). i–iv) Authentic standards of chanoclavine-I aldehyde (3; i), agroclavine (5; ii), festuclavine (4; iii), and cycloclavine (6, iv). v, vi) Reaction products from the incubation of Aj_EasA, Aj_EasG, and Aj_EasH with chanoclavine-I aldehyde (3) and cofactors: v) by-product, festuclavine (4); vi) predominant enzymatic product, cycloclavine (6).

Cycloclavine (**6**) has been observed in only one species of filamentous fungus, *Aspergillus japonicus*.[3a] Inspection of the *A. japonicus* genome revealed a 16.8 kbp biosynthetic cluster containing eight genes (for the organization of the cluster, see Figure S1 in the Supporting Information),[4] seven of which (*dmaW*, *easF*, *easE*, *easC*, *easD*, *easA*, *easG*) are homologous to genes previously implicated in the biosynthesis of festuclavine (**4**) or agroclavine (**5**) in other filamentous fungi (Figure 1 a).[2] We set out to validate whether this cluster was responsible for cycloclavine biosynthesis by reconstitution of the eight genes in *S. cerevisiae*. Synthetic genes were used for the construction of all strains, and a combination of GPD1 promoter/CYC1 terminator, PGK1 promoter/ADH2 terminator, PDC1 promoter/FBA1 terminator, TEF1 promoter/ENO2 terminator, and TEF2 promoter/PGI1 terminator was used for the expression cassettes (see the Supporting Information).[5,6] A previously reported *S. cerevisiae* strain produces the early ergot-alkaloid intermediate chanoclavine-I (**2**) from the biosynthetic genes *dmaW* (*A. japonicus*), *easF* (*Aspergillus fumigatus*), *easE* (*A. japonicus*), and *easC* (*A. japonicus*) in titers of 0.75 mg L^−1^.[5] This relatively low level was associated with the failure of *N*-methyl-4-dimethylallyl-l-tryptophan (*N*-Me-DMAT, **1**) to be converted efficiently into chanoclavine-I (**2**; Figure 1 a). Qualitative increases in the levels of chanoclavine-I (**2**) were observed in response to growth at decreasing temperatures and may correspond to improved folding of the proteins responsible for the conversion of *N*-Me-DMAT (**1**) into chanoclavine-I (**2**; see Figure S2). The increase in chanoclavine-I production provided a basis for extending the ergot-alkaloid pathway in yeast.

We transformed this chanoclavine-I-producing strain with combinations of expression vectors carrying the remaining genes of the *A. japonicus* cluster (*easD*, *easA*, *easG*, and *easH*; Figure 1 a). When *easD*, *easA*, and *easG* were added, festuclavine (**4**) was observed (see Figure S3), which was not unexpected, since festuclavine (**4**) is produced by homologues of these seven genes found in other filamentous fungi, such as *A. fumigatus*.[7–10] Gratifyingly, when *easH*, for which no role was previously known, was added along with *easD*, *easA*, and *easG*, the predominant product was cycloclavine (**6**), thus clearly demonstrating that *easH* is necessary for cycloclavine biosynthesis (Figure 2; see also Figure S4). Notably, concomitant production of festuclavine (**4**) was also observed. Since both cycloclavine (**6**) and festuclavine (**4**) have been isolated from *A. japonicus*,[3a] we hypothesize that this gene cluster produces a mixture of these two compounds in the native host, though how this ratio is impacted by environmental conditions is unknown. To assess whether increased amounts of EasH would impact the festuclavine/cycloclavine ratio, we constructed a strain carrying the entire eight-gene cluster supplemented with additional copies of *easH* from plasmid vectors. We observed a clear gene-dose-dependent increase in the ratio of cycloclavine (**6**) to festuclavine (**4**) as the copy number of *easH* increased (see Figure S4).

**Figure 2:**
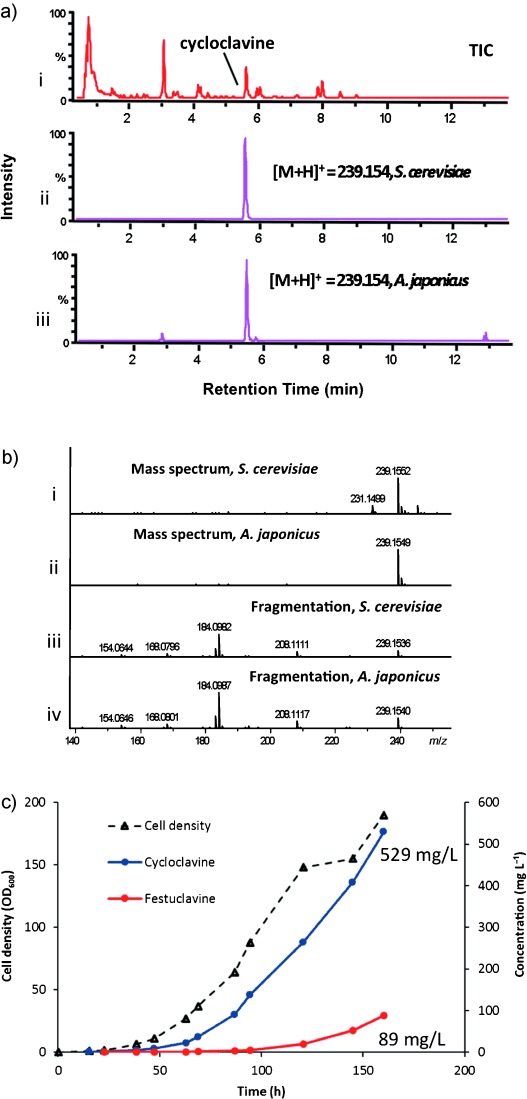
Production of cycloclavine (6) in yeast. a) i) Total ion current (TIC) chromatogram from *S. cerevisiae* expressing the entire cycloclavine cluster; ii) chromatogram showing the compound produced by an engineered strain of *S. cerevisiae* with an extracted [*M*+H]^+^ value of 239.154; iii) chromatogram showing cycloclavine (6) from *Aspergillus japonicus* with the same mass. b) Extracted ion chromatograms ([*M*+H]^+^=239.154) and mass fragmentations of compounds from i, iii) *S. cerevisiae*, expressing the cycloclavine cluster, and ii, iv) the native producer *A. japonicus*. c) Fermentation of *S. cerevisiae*, with the production of a final titer of 529 mg L^−1^ of cycloclavine (6) and 89 mg L^−1^ of festuclavine (4).

Complex ergot alkaloids constitute a rich source of biologically active compounds, and a robust production platform for these molecules will improve accessibility and prospects for commercial application. Whereas chanoclavine-I (**2**) has been successfully reconstituted in *S. cerevisiae* as well as another heterologous host, *Aspergillus nidulans*,[5,11] more derivatized ergot alkaloids have not been subject to such efforts. To examine whether we could produce the complex ergot alkaloid cycloclavine (**6**) in high yields in *S. cerevisiae*, we integrated multiple copies of cycloclavine-pathway genes into the genome of the commonly used yeast strain S288C. The best strain had an additional copy of *easG*, two additional copies of *dmaW* and *easD*, and three additional copies of *easC* and *easH*. Moreover, additional copies of the host genes *pdi1* (protein disulfide isomerase) and *fad1* (FAD synthetase) were also included to assist in the production of the disulfide- and flavin-containing enzyme EasE (see Table S1 in the Supporting Information). This strain resulted in the production of cycloclavine (**6**) with a final concentration of 529 mg L^−1^ in the growth medium when fermentation was carried out for 160 h in a 1 L fermenter and a fed-batch regime was used with restricted feeding starting after 40 h (Figure 2). Additionally, the strain produced festuclavine (**4**) at a final concentration of 89 mg L^−1^. The excellent production level for this eight-step pathway highlights the prospects for large-scale heterologous expression of the ergot-alkaloid class of natural products. The structure of cycloclavine (**6**) was fully characterized by ^1^H NMR, ^13^C NMR, ^1^H,^1^H-ROESY, and ^1^H,^13^C-HMBC spectroscopic experiments (see Table S2 and Figures S5–S7).

The selective production of cycloclavine (**6**) versus festuclavine (**4**) requires an understanding of the enzyme mechanism. As a starting point to explore the unusual reaction(s) that generate cycloclavine (**6**), we assessed whether EasH, which is annotated as an Fe^II^/2-oxoglutarate-dependent dioxygenase, could be assayed in vitro. Upon the incubation of chanoclavine-I aldehyde (**3**) with enzymes heterologously expressed and purified from *Escherichia coli* (EasA, EasG) and yeast (EasH), along with Fe^II^, NADP^+^, NADPH, ascorbic acid, and 2-oxoglutarate, we observed the formation of cycloclavine (**6**) as evidenced by the exact mass and coelution with an authentic cycloclavine standard (Figure 1 b, iv and vi; see Figures S8–S10). Product formation increased with increasing reaction time and substrate concentration (see Figures S11–S15). Festuclavine (**4**) was also observed as a by-product in the in vitro enzymatic reaction of EasA/G/H (Figure 1 b, v), and the reaction of EasA and EasG with chanoclavine-I aldehyde (**3**) in the absence of EasH yielded festuclavine (**4**; see Figure S16). Manipulation of the EasA/G/H ratio yielded variation in the ratio of cycloclavine (**6**) to festuclavine (**4**; see Figure S17), as was observed when additional copies of *easH* were expressed in the yeast production platform. The highest amount of **6** was observed with a 1:1:10 ratio of enzymes (see Figure S17), whereas the highest concentration of **4** was observed when EasA was present in tenfold excess (see Figure S17). When Fe^II^ or α-ketoglutarate was removed from the reaction, no cycloclavine (**6**), only festuclavine (**4**), was observed, thus suggesting that these cofactors are necessary for cyclopropyl formation (see Figure S10). Surprisingly, when EasH was subjected to more than one purification step, it was inactive unless nicotinamide adenine dinucleotide phosphate (NADP^+^) was added (see Figure S10 d). EasH appears to weakly copurify with NADP^+^, which we speculate may be required to stabilize the enzyme, but this cofactor is lost after more than one enzyme-purification step (see Figure S18). The absence of NADPH, which is required by reductase EasG, did not inhibit the reaction, thus suggesting that NADP^+^ is reduced in situ under the enzyme assay conditions to NADPH.

Although both in vitro and in vivo assays indicated that EasA, EasG, and EasH convert chanoclavine-I aldehyde (**3**) into cycloclavine (**6**), the individual roles of these enzymes remained unclear. The catalytic activities of EasA and EasG have been previously established in other ergot-alkaloid pathways. EasA is a flavin-containing enzyme that either reduces or isomerizes (Figure 1 a) the double bond of chanoclavine-I aldehyde (**3**),[10,12] thus allowing formation of the six-membered D ring. The resulting iminium species (Figure 1 a) is reduced by the NADPH-dependent reductase EasG to yield either festuclavine (**4**; reductive EasA) or agroclavine (**5**; isomerase EasA).[9,10,12,13]

EasH, along with the appropriate cofactors, was incubated with festuclavine (**4**), the product of EasA and EasG. However, only starting material was observed, thus indicating that festuclavine (**4**) is not a substrate for EasH (see Figure S19). EasH was also incubated with agroclavine (**5**) and chanoclavine-I aldehyde (**3**), but in both cases, no formation of a new product, or disappearance of the starting material, was observed (see Figure S19). These observations strongly suggest that EasH acts upon a reaction intermediate that occurs during the course of the EasA/EasG-catalyzed transformation. In support of this hypothesis, when EasH and EasA were co-incubated along with all substrates and cofactors, the formation of an intermediate with a mass (M^+^) of 237 was observed (see Figure S20). Although this compound could not be isolated, the mass is consistent with the structure of intermediate **10** (Scheme 1). When EasG was added, the compound with the mass corresponding to intermediate **10** disappeared, and the formation of cycloclavine (**6**) was observed (see Figure S20). Additionally, the presence of intermediate **8**, in the presence of EasA (fivefold excess), EasG, and EasH, was validated by deuterium labeling through selective reduction with sodium cyanoborodeuteride (see Figures S21 and S22).

**Scheme 1:**
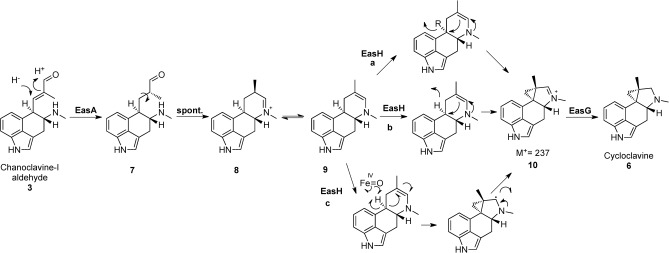
Proposed mechanisms of cycloclavine formation. EasA is an Old Yellow Enzyme homologue, EasG is an NADPH-dependent reductase, and EasH is annotated as an α-ketoglutarate-dependent, non-heme iron oxygenase; R=OH or Cl. a) EasH hydroxylates or halogenates 9. b) EasH abstracts a hydride ion from 9. c) EasH abstracts a hydrogen atom from 9.

EasH has two well-characterized homologues, both of which are Fe^II^- and 2-oxoglutarate-dependent enzymes that catalyze hydroxylation reactions (see Figure S8). Phytanoyl-CoA 2-hydroxylase hydroxylates an aliphatic carbon atom of a fatty-acid derivative,[14] and EasH from *Claviceps purpurea* hydroxylates the late-stage ergot intermediate dihydroergotamam.[15] Recently, Robinson and Panaccione reported the expression of an *Epichloë sp. easH* gene in a fungal strain that produces agroclavine (**5**),[16] though the catalytic function of this homologue has not yet been elucidated.

For cyclopropyl formation, we eliminated any mechanism in which EasH acts directly on festuclavine (**4**), agroclavine (**5**), or chanoclavine-I aldehyde (**3**), since such activity is inconsistent with the results of the biochemical assays. More consistent would be the reduction of chanoclavine-I aldehyde (**3**) by EasA, as occurs in the biosynthesis of festuclavine (**4**), to yield intermediate **8**, which could then undergo imine/enamine tautomerization to yield **9**, the substrate for EasH (Scheme 1). If EasH acts as a hydroxylase, as does its characterized homologues,[14,15] EasH could hydroxylate iminium species **8**. Water could then be eliminated to form the cyclopropyl group, and the product could be reduced by EasG (Scheme 1a). Notably, the sequences of Fe^II^/2-oxoglutarate-dependent halogenases and hydroxylases are similar, which raises the possibility that EasH could function as a halogenase, in which case a halide ion would serve as a leaving group in this mechanism (Scheme 1 a). However, iron-dependent halogenases typically contain the consensus sequence HQA,[17,18] whereas the corresponding sequence in EasH is HRE (see Figure S8), a sequence that is more consistent with hydroxylase enzymes. EasH could also catalyze the formation of the cyclopropyl ring by abstraction of the benzylic hydride by NADP^+^, followed by cyclization (Scheme 1b). Alternatively, abstraction of the benzylic hydrogen atom could be catalyzed by the iron cofactor, followed by ring formation by radical cyclization (Scheme 1c). Although the closest known EasH homologues have demonstrated hydroxylase activity, unless additional reaction intermediates in cycloclavine biosynthesis can be trapped, the detailed mechanism by which EasH catalyzes cycloproponation remains speculative. Nevertheless, it is clear that the oxidase EasH is directly responsible for the formation of the cyclopropyl group and uses one of the reaction intermediates formed by EasA as a substrate.

In summary, cycloclavine (**6**) was produced in high levels through yeast fermentation. Although the production of the intermediate chanoclavine-I has been reported previously,[5,11] cycloclavine (**6**) is the first downstream ergot alkaloid to be successfully reconstituted in excellent yields. EasH was identified as the enzyme responsible for switching the pathway from the biosynthesis of festuclavine (**4**) or agroclavine (**5**) to that of cycloclavine (**6**). Although the mechanistic details of EasH require further exploration, it is clear that EasH generates a cyclopropyl moiety through an oxidative mechanism by intercepting one of the reaction intermediates generated during the conversion of chanoclavine-I aldehyde (**3**) into cycloclavine **6**. This study lays the foundation for more translational applications of the ergot alkaloids and has revealed another example of the enzymatic formation of a cyclopropyl group in nature.

## Notes

Supporting information for this article, including all experimental details, is available on the WWW under http://dx.doi.org/10.1002/anie.201410002.
