# Supplementary material for: Discovery and Reconstitution of the Cycloclavine Biosynthetic Pathway—Enzymatic Formation of a Cyclopropyl Group
Source: Angew Chem Int Ed Engl. 2015 Feb 25;54(17):5117–21. doi: 10.1002/anie.201410002 (PMC4471609; doi:10.1002/anie.201410002)
Supplement: Supplementary file 1 [file anie0054-5117-sd1.pdf]

Angewandte Chemie
Eine Zeitschrift der Gesellschaft Deutscher Chemiker

Supporting Information

Discovery and Reconstitution of the Cycloclavine Biosynthetic Pathway—Enzymatic Formation of a Cyclopropyl Group**

Dorota Jakubczyk, Lorenzo Caputi, Anaëlle Hatsch, Curt A. F. Nielsen, Melanie Diefenbacher, Jens Klein, Andrea Molt, Hartwig Schröder, Johnathan Z. Cheng, Michael Naesby, and Sarah E. O'Connor**

anie_201410002_sm_miscellaneous_information.pdf

Table of Contents

SI–1 – Methods.....3
SI-1.1 General materials and methods.....3
SI-1.2 DNA and protein sequence analysis.....3
SI-1.3 Construction and integration of gene expression cassettes for *in vivo* studies.....3
SI-1.4 Shake flask culture conditions.....4
SI-1.5 Fed-batch fermentation conditions.....5
SI-1.6 Cloning, overexpression, and purification of *Aspergillus japonicus* EasD, EasA, EasG and EasH for *in vitro* studies.....6
SI-1.7 Cloning, overexpression, and purification of *Aspergillus fumigatus* EasA, EasG.....9
SI-1.8 *In vitro* endpoint assays for EasA, EasG and EasH.....10
SI-1.9 Analytical procedures for *in vivo* experiments.....10
SI-1.10 Purification of cycloclavine **6** and festuclavine **4** from yeast cultures.....11
SI-1.11 Mass spectrometry (*in vitro* assays) 11
SI-1.12 Nuclear magnetic resonance (NMR) spectroscopy.....12
SI-2 Supporting Figures.....13
Figure S1 Organization of the *Aspergillus japonicus* cycloclavine **6** gene cluster.....13
Figure S2 A yeast strain expressing the biosynthetic pathway to chanoclavine-I **2**.....14
Figure S3 A chanoclavine-I **2** producing yeast was used for ectopic expression of combinations of ergot alkaloid genes.....15
Figure S4 The ratio between cycloclavine **6** and festuclavine **4** production depends on the copy number of *easH.*.....16
Figure S5 NMR data (^{1}H- and ^{13}C-NMR chemical shifts).....17
Figure S6 Key HMBC correlations of cycloclavine **6**.....18
Figure S7 Key ROESY correlations of cycloclavine **6**.....18
Figure S8 Alignment of EasH (*A. japonicus*) and homologs.....19
Figure S9 SDS-PAGE of heterologously expressed proteins.....20
Figure S10 *In vitro* assays with varying co-factor combinations.....21
Figure S11 Standard curve for cycloclavine **6** concentration and peak area response observed by LC-MS.....22
Figure S12 Time course of EasH assays.....22
Figure S13 Concentration of cycloclavine **6** increases with increasing EasH concentration and with time.....23
Figure S14 Cycloclavine **6** formation dependence on concentration of chanoclavine-I aldehyde **3** and time.....24
Figure S15 The pH optimum of cycloclavine **6** formation by EasA, EasG and EasH (*A. japonicus*) was determined to be pH = 7.....25
Figure S16 Assays with chanoclavine-I aldehyde**3** and different enzyme combinations.....26
Figure S17 Cycloclavine **6** and festuclavine **4** production with different ratio of enzymes.....27
Figure S18 UV spectra of EasH.....28
Figure S19 Assays with EasH (no EasA or EasG).....29
Figure S20 Detection of intermediate in *in vitro* cycloclavine biosynthetic reactions.....30
Figure S21 Intermediates observed in assays with enzymes cloned from both *A. japonicus* and *A. fumigatus*.....31
Figure S22 Trapping of iminium ion.....32
SI-3 Supporting Tables.....33
Table S1. Integration constructs inserted into *S. cerevisiae* S288c chromosomal DNA.....33
Table S2. ^{1}H-NMR and ^{13}C-NMR shifts of cycloclavine **6**.....34
Table S3. Exact Masses of compounds were determined by electrospray (ESI) (operated in positive mode) high resolution mass spectrometry (MS) 35
Table S4. Operating conditions for the fed-batch fermentation.....35
Table S5. Composition of the “SC” medium for fermentation.....36
Table S6. Composition of feed solution, using NH_4OH as nitrogen source.....36
Table S7. Composition of the stock trace metal solution.....37
Table S8. Composition of the stock vitamin solution.....37
References for Supporting Information......37

SI–1 - Methods

SI-1.1 General materials and methods

Chanoclavine-I aldehyde **3**, festuclavine **4** and agroclavine **5** were prepared or obtained as previously reported.[1] Acetonitrile was of LC-MS grade and was purchased from Sigma-Aldrich (St. Louis, MO, USA). All other chemicals and solvents were of analytical grade and used as purchased. All buffers and solutions were prepared in Milli-Q water.

Primers for cloning were synthesized by Integrated DNA Technologies (USA) and sequencing of the constructs was performed by Sourcebioscience (UK). Platinum Taq DNA polymerase (Life Technologies) or iProof High Fidelity DNA polymerase (BioRad) was used for PCR amplification of the genes. Restriction enzymes and T4 DNA ligase were purchased from New England Biolabs (USA). A Varian Cary 50 Bio Scanning Spectrometer was used to acquire UV-Vis spectra.

SI-1.2 DNA and protein sequence analysis

Computer-aided sequence analysis was done using Vector NTI 9.1.0 software (Invitrogen Corp. 2004) and the free online software FGENESH (http://linux1.softberry.com/berry.phtml), GENSCAN (http://genes.mit.edu/GENSCAN.html), and the NCBI server (http://www.ncbi.nlm.nih.gov). Signal peptides were predicted using the SignalP 4.0 tool[2].

SI-1.3 Construction and integration of gene expression cassettes for *in vivo* studies

Fungal genes used for *in vivo* expression were predicted from the genomic DNA cluster of *Aspergillus japonicus*[3], except for the *easF* from *Aspergillus fumigatus* which was based on public sequence information (GenBank:XM_751050). Genes were synthesized, with yeast codon optimization, by DNA2.0 Inc., Menlo Park, CA, USA or GeneArt AG, Regensburg, Germany. The *S. cerevisiae* genes *pdi1* and *fad1* were amplified by PCR directly from genomic DNA.

For expression, all genes were cloned into expression vectors based on pRS313, pRS314, pRS315, and pRS316[4,5]. These vectors were provided with expression cassettes, comprising promoters and terminators amplified by PCR from yeast genomic DNA as previously described[5]. Five expression cassettes containing 1) a *GPD1* promoter and a *CYC1* terminator (G/C), 2) a *PGK1* promoter and an *ADH2* terminator (P/A), 3) a *PDC1* promoter and an *FBA1* terminator (P/F), 4) a *TEF1* promoter and an *ENO2* terminator (T/E), and 5) a *TEF2* promoter and a *PGI1* terminator (T/P) were constructed in the pRS vectors. Integration vectors were constructed, based on these expression cassettes, as previously described for *YORWΔ22*[5,6]. Four additional constructs were made based on the integration sites *YHRCΔ14, YMRWΔ15, YNRCΔ9,* and*YPRC τ3,* described by Flagfeldt et al.[6], and each construct was equipped with 3-5 expression cassettes.

The yeast strain used for cycloclavine **6** production was the *Saccharomyces cerevisiae* S288c, Mat α, NCYC3608, obtained from the National Collection of Yeast Cultures, Norwich, U.K. This basic strain has the KanMX antibiotic marker inserted into the Ura3 locus, and a non-functional HO locus[7]. This strain was subjected to 5 rounds of transformation, integrating a total of 21 gene expression cassettes (excluding markers) into the yeast genome to yield the cycloclavine producing strain (Table S1).

SI-1.4 Shake flask culture conditions

Engineered yeast strains were grown in standard SC broth with 2% glucose, minus relevant amino acids (ForMedium, Hunstanton, U.K.). Cultures for analysis were started from re-streaked, single colonies. These were grown overnight in standard SC broth, and then diluted to an optical density, at 600 nm, of 0.1 to start the main culture. Unless otherwise stated, main cultures were grown at 20°C with constant shaking at 150 rpm, 5 cm amplitude, for 72 hours in 250 mL shake flasks containing 25 mL medium.

SI-1.5 Fed-batch fermentation conditions

Production of cycloclavine **6** and festuclavine **4** in yeast appeared to be coupled to biomass production, and hence the fermentation process was a fed-batch that aimed to produce high biomass. Therefore, a conventional feeding regime was chosen and aeration and stirring regimes were aimed to avoid fermentative metabolism and minimize glucose accumulation as well as ethanol- and acetate formation.

The process was started as a batch using synthetic complete medium (SC), after which a feed was started that contained glucose, salts, vitamins, trace metals and amino acids. The platform used was a Multifors2 from INFORS HT, Binningen, Switzerland, with a working volume of 1 L. The starting volume was 0.32L, then feed was added using a predefined 2-phase exponential feed regime followed by a constant feed regime. The final Cell-Dry-Weight (CDW) reached was ca. 57 g/L. The concentration of cycloclavine **6** obtained in the clarified broth (i.e. after removing of cells) was ca. 529 mg/L. Moreover, ca. 89 mg/L of festuclavine **4** were detected as well.

The fermenter was equipped with two Rushton six-blade disc turbines. Air was used for sparging the fermenters. Temperature, pH, agitation, and aeration rate were controlled throughout the cultivation. The temperature was maintained at 20 °C. The pH was kept at 5.85 by automatic addition of 2M NH_4OH during the starting batch phase and of 5M NH_4OH during the feeding stage of the fermentation. The stirrer speed was set to 650 rpm and the aeration rate was kept to 1.0 vvm in order to prevent the Dissolved Oxygen (DO) dropping below 20%. The operating conditions used in the fermentation are summarized in Table S4.

For inoculation of the fermenter a 1-stage seed train was used. Seed cultures were prepared by inoculating 50 mL of medium in a 500-ml shake flask with 4 baffles (indents) to a starting OD_{600} of ca. 0.4, using freshly grown colonies from an YPD plate. The seed medium consisted of SC-medium with 20 g/L glucose (Table S5). The shake flasks were

placed on a shaking table with amplitude of 25 mm at 250 rpm at 20 °C. The cells were grown into exponential phase until, after ~23 h., the OD_{600} was ca. 3 (CDW ca. 0.9 g/L).

Prior to inoculation, an amount of the batch-medium in the fermenter equivalent to the amount of inoculum was removed and an aliquot of 10 mL of the seed culture was used for inoculation of the fermenter to a final volume of 0.32 L and a starting OD_{600} of ca. 0.1 (CDW ca. 0.03 g/L). Fermentation was started as a batch of ca. 40 hours, with a starting volume of 0.32 L of SC-medium with 20 g/L glucose (Table S5).

After ca. 40 hours, a feed was started that contained a mixture of glucose, vitamins, trace-metals and salts, and was furthermore enriched with amino acids (Table S6). During said feeding phase, ammonium hydroxide (5M NH_4OH) was used both as the nitrogen source and the base to control pH. Tables S7 and S8 list the compositions of the stock solutions of trace metals and vitamins, respectively.

The estimated total addition of feed was ca. 68 mL in 120 hours, according to a feed regime consisting of i) an exponential feeding phase of 30 hours, during which ca. 16 mL of feed were added, followed by ii) a second exponential feeding phase also of 30 hours during which ca. 15 mL of feed were added, and iii) a final constant feeding phase of 60 hours during which ca. 37 mL of feed were added.

SI-1.6 Cloning, overexpression, and purification of *Aspergillus japonicus* EasD, EasA, EasG and EasH for *in vitro* studies

The coding sequences (CDS) of EasD, EasA, EasG, and EasH were predicted from the genome sequence, using free on-line prediction tools, and synthesized with yeast codon optimization. The genes were cloned into plasmids pRS pRS313-316, which had been equipped with yeast promoters and terminators (see above). These vectors carry different auxotrophic markers allowing simultaneous expression in the yeast cell.

For heterologous protein expression, *easA* was amplified using the following primer pair: forward primer 5'-GATTCATATGACCATCGATCCATCT-3' (NdeI site underlined); reverse

primer 5’-TTCCAAGCTTTTAACCTTGACAAATAGAACC-3’ (HindIII site underlined) and ligated into the NdeI/ HindIII cloning site in pET28a(+) (Novagen) expression vector. Expression of EasA (Aj) was carried out in LB medium containing kanamycin (50 μg/mL) and chloramphenicol (34 μg/mL). *E. coli* Rosetta (DE3) pLysS cells (Invitrogen) harbouring the construct were grown to an OD_{600} of 0.7 at room temperature and then cooled to 15°C for 1 h prior to induction with IPTG (1 mM). Cultures were grown for 16 hours at 15°C prior to harvesting by centrifugation at 3500 x g for 15 min. Cells were re-suspended in 20 mM Tris-HCl buffer pH 8.0 containing 300 mM NaCl, 10% (v/v) glycerol and protease inhibitors (Roche) and lysed by cell disruptor. Cellular debris was pelleted by centrifugation (23,000 x g for 30 min). Crude EasA enzyme was purified by affinity chromatography on Ni-NTA agarose (Qiagen). The yield of EasA was estimated to be 2 mg per litre of culture as measured by bicinchoninic acid (BCA) assay. Fractions containing pure EasA, as demonstrated by SDS-PAGE, were collected and dialyzed against dialysis buffer (50 mM K_2HPO_4, 100 mM NaCl, 10% (v/v) glycerol, pH = 7.0) (Figure S9).

For heterologous protein expression, *easG* was amplified using the following primers: forward primer 5’-GATTCATATGACCATCTTGGTTTTG-3’ (NdeI restriction site underlined); reverse primer5’-GGAAAAGCTTTTACAACCAATGTTCTCTGT-3’ (HindIII restriction site underlined) and ligated into the NdeI / HindIII cloning site in pet28a(+) (Novagen) expression vector. Expression was carried out in LB media containing kanamycin (50 μg/mL) and chloramphenicol (34 μg/mL). *E. coli* Rosetta (DE3) pLysS cells (Invitrogen) harbouring the construct were grown to an OD_{600} of 0.7 at room temperature and then cooled to 15°C for 1 h prior to induction with IPTG (0.1 mM). Cultures were grown for 16 hours at 15°C prior to harvesting by centrifugation at 3500 x g for 15 min. Cells were re-suspended in 50 mM Tris-HCl buffer pH 8.0 containing 50 mM glycine, 500 mM NaCl, 5% (v/v) glycerol, 20 mM imidazole and protease inhibitors (Roche) and lysed by cell disruptor. Cellular debris was pelleted by centrifugation (23,000 x g for 30 min). The supernatant was loaded onto a

HisTrap FF 5 mL column at a flow rate of 4 mL/min. The column was then washed at 4 mL/min with buffer A until the absorbance at 280 nm reached the baseline value. EasG was step eluted using 50 mM Tris-HCl buffer pH 8.0 containing 50 mM glycine, 500 mM NaCl, 5% (v/v) glycerol and 500 mM imidazole at 4 mL/min. The eluted enzyme was then concentrated to approximately 400 μL volume and injected (2 x 200 μL) onto a Superdex 200 10/300 GL column (GE Healthcare) equilibrated with 50 mM phosphate buffer pH 7.0 containing 150 mM NaCl. The gel filtration chromatography was performed on an Akta purifier (GE Healthcare) at 0.6 mL/min using 50 mM phosphate buffer pH 7.0 as mobile phase. Elution was followed at 280 nm and 0.5 mL fractions were collected. The yield of EasG was estimated to be 1.2 mg per litre of culture as measured by BCA assay. Fractions containing pure EasG, as demonstrated by SDS-PAGE, were collected and dialyzed against dialysis buffer (50 mM K_2HPO_4, 100 mM NaCl, 10% (v/v) glycerol, pH = 7.0) (Figure S9).

For heterologous protein expression, *easH* was amplified from a pRS vector (see above) and cloned into pEVE1914. This yeast expression vector provided an N-terminal FLAG-tag for protein purification, and allowed expression of the fusion protein from a Cup1 promoter. Expression of EasH was carried out in synthetic complete medium (SC) without uracil. *Saccharomyces cerevisiae* InvSci1 cells were grown to an OD_{600} of 0.8 (2 days) prior to induction with copper(II) sulfate (300 μM) and grown for 16 hours at 20°C prior to harvesting. Cells were re-suspended in buffer (50mM K_2HPO_4, 100 mM NaCl, 10% (v/v) glycerol, pH = 7.0) and lysed by cell disruptor. Cellular debris was pelleted by centrifugation (23,000 x g for 0.5 hour). Crude EasH enzyme was purified by FLAG-tag purification. The eluted enzyme was then concentrated to approximately 400 μL volume and injected (2 x 200 μL) onto a Superdex 200 10/300 GL column (GE Healthcare) equilibrated with 50 mM phosphate buffer pH 7.0 containing 150 mM NaCl. The gel filtration chromatography was performed on an Akta purifier (GE Healthcare) at 0.6 mL/min using 50 mM phosphate buffer pH 7.0 as mobile phase. Elution was followed at 280 nm and 0.5 mL fractions were collected. The yield of

EasH was estimated to be 2 mg per litre of culture as measured by BCA assay. Fractions containing EasH, as demonstrated by SDS-PAGE, were collected and dialyzed against dialysis buffer (50 mM K_2HPO_4, 100 mM NaCl, 10% (v/v) glycerol, pH = 7.0) (Figure S9).

SI-1.7 Cloning, overexpression, and purification of *Aspergillus fumigatus* EasA, EasG

Cloning of *easA* from *A. fumigatus* into pET-28a(+) (Novagen) was previously reported[1]. Expression was carried out in LB media with kanamycin (50 µg/mL). *E. coli* SlouBL21 cells were grown to an OD_{600} of 0.7 prior to induction with IPTG (1 mM) and grown for 16 hours at 15°C prior to harvesting. Cells were re-suspended in buffer (20mM Tris-HCl, 300 mM NaCl, 10% (v/v) glycerol, pH = 8.0) and lysed by cell disruptor. Cellular debris was pelleted by centrifugation (23,000 x g for 0.5 hour). Crude EasA enzyme was purified by Ni-NTA agarose (Qiagen). The yield of EasA was estimated to be 1.2 mg per litre of culture as measured by BCA assay. Fractions containing pure EasA, as demonstrated by SDS-PAGE, were collected and exchanged with dialysis buffer (50 mM K_2HPO_4, 100 mM NaCl, 10% (v/v) glycerol, pH = 7.0) (Supplementary Fig. S9).

Cloning of *easG* from *A. fumigatus* into pET-24a(+) (Novagen) was previously reported.[8] Expression was carried out in LB medium (supplemented with 1% glucose) with kanamycin (50 µg/mL) and chloramphenicol (34 µg/mL). *E. coli* Rosetta (DE3) pLysS cells (Invitrogen) were grown to an OD_{600} of 0.8, then transferred to 15°C for 1 h prior to induction with IPTG (1 µM) and grown for 16 hours at 15°C prior to harvesting. After centrifugation at 3500 x g for 15 min, the cells were re-suspended in 50 mM Tris-HCl buffer pH 8.0 containing 50 mM glycine, 500 mM NaCl, 5% (v/v) glycerol, 20 mM imidazole and protease inhibitors (Roche) and lysed by cell disruptor. Cellular debris was pelleted by centrifugation (23,000 x g for 30 min). The supernatant was loaded onto a HisTrap FF 5 mL column at a flow rate of 4 mL/min. The column was then washed at 4 mL/min with buffer A until the absorbance at 280 nm reached the baseline value. EasG was step eluted using 50 mM Tris-HCl buffer pH 8.0 containing 50 mM glycine, 500 mM NaCl, 5% (v/v) glycerol and 500 mM imidazole at 4

mL/min. The eluted enzyme was then concentrated to approximately 400 µL volume and injected (2 x 200 µL) onto a Superdex 200 10/300 GL column (GE Healthcare) equilibrated with 50 mM phosphate buffer pH 7.0 containing 150 mM NaCl. The gel filtration chromatography was performed on an Akta purifier (GE Healthcare) at 0.6 mL/min using 50 mM phosphate buffer pH 7.0 as mobile phase. Elution was followed at 280 nm and 0.5 mL fractions were collected. The overall yield of EasG was estimated to be 1 mg per litre of culture as measured by BCA assay. Fractions containing pure EasG, as demonstrated by SDS-PAGE, were collected and exchanged with dialysis buffer (50 mM K_2HPO_4, 100 mM NaCl, 10% (v/v) glycerol, pH = 7.0) (Figure S9).

SI-1.8 *In vitro* endpoint assays for EasA, EasG and EasH

In vitro assays used EasA, EasG and EasH purified as described above. Proteins were taken as frozen aliquots stored at -80 °C. Enzymes could be stored without detectable loss of activity for at least three months. A standard endpoint assay incubated EasA (0.5 µM, final concentration), EasG (0.5 µM, final concentration), EasH (0.5 µM, final concentration), chanoclavine-I aldehyde **3** (5 µM, final concentration), $NADP^+$ (500 µM, final concentration), 100 µM α-ketoglutaric acid, 100 µM L-ascorbic acid and 10 µM $FeSO_4$*$7H_2O$ in 100 mM K_2HPO_4 buffer (pH = 7.0, Figure S15) at 25°C for 30 minutes. All additional *in vitro* assays with varying time, co-factor and enzyme concentrations are specified in detail in the corresponding figure legends below. Aliquots (10 µL) were quenched by dilution in 40 µL of 0.1% formic acid (in water) and analyzed by UPLC-MS/MS as described in section SI-1.11.

SI-1.9 Analytical procedures for *in vivo* experiments

For analysis, yeast cultures were spun down for 10 min at 1000 × g. The pellet and the supernatant were separated. Without further purification, 5 µl of supernatant were injected in a UPLC-TOF (Waters AcquityTM Ultra Performance LC, Waters, Milford, Mass., USA) coupled to a micrOTOF-Q II (Bruker Daltonik GmbH, Bremen, Germany). Stationary phase column was an Acquity UPLC® Bridged Ethyl Hybrid (BEH) C18; 1.7 µm; 2.1×100 mm.

Liquid chromatography used mobile phases of H_2O + 0.1% formic acid (A), and acetonitrile + 0.1% formic acid (B), in a linear gradient of 1% to 100% B in 12 min. The column was washed for 5 min in 100% B, and then equilibrated for 2 min in 1% B. Detection of compounds was done by a photo diode array using the following parameters: λ range: 210 nm to 500 nm. Resolution: 1.2 nm. Sampling rate: 5 points/s. ELSD parameters: gain 50, gas pressure 40 psi, nebulizer mode: heating, power level: 80%, drift tube: 80°C. TOF parameters: Source: End Plate Offset: -500V. Capillary: -4500V. Nebulizer: 1.6 bar. Dry gas: 8.0 L/min. Dry temperature 180°C. Scan mode: MS Scan. Mass range: from 80 to 1000 Da.

SI-1.10 Purification of cycloclavine 6 and festuclavine 4 from yeast cultures

Cultures were spun down for 10 min at 1000 × g. The supernatant was adjusted to pH=10 with 10M NaOH and extracted by liquid/liquid extraction with an equal volume of ethyl acetate. The crude extract was dried under vacuum and reconstituted with dimethyl sulfoxide (DMSO) to a concentration of 100 mg/mL and then purified on a preparative HPLC system (Waters, Milford, Mass, USA). Stationary phase was an XBridgeTM preparative C18, 5 µm, 19×250 mm column. Liquid chromatography used mobile phases of H_2O + 0.1% trifluoroacetic acid (A), and acetonitrile + 0.1% trifluoroacetic acid (B), in a linear gradient of 1% to 30% B in 40 min. The column was washed for 5 min in 100% B, and then equilibrated for 10 min in 1% B. Fractions were collected every 2 min and analyzed as above. Fractions containing the purified analyte were pooled and dried under vacuum.

SI-1.11 Mass spectrometry (*in vitro* assays)

Ultraperformance liquid chromatography was performed on a Waters Acquity UPLC system consisting of a binary pump, an online vacuum degasser, an autosampler, and a column compartment. Separation of the analytes was achieved on a Waters Acquity Ultra Performance BEH C18 column with 1.7 µm particle size, 2.1 x 50 mm, kept at 30 °C. Mobile phase A was water containing 0.1% formic acid; mobile phase B was acetonitrile containing 0.1% formic acid. The flow was 0.6 mL/min, and the gradient profile was 0 min, 5% B; from

0 to 5 min, linear gradient elution to 30% B; from 5 to 7 min, back to initial conditions of 95% B. The injection volume of both the standards and the samples was 2 µL. After each injection, the needle was rinsed with 600 µL of weak wash solution (water/acetonitrile, 90:10) and 200 µL of strong wash solution (acetonitrile/water, 90:10). Samples were kept at 10 °C during the analysis.

Mass spectrometry detection was performed on a Waters Xevo TQ-S instrument equipped with an electrospray (ESI) source operated in positive mode. Capillary voltage was 3.8 kV; the source was kept at 150 °C; desolvation temperature was 200 °C; cone gas flow, 150 L/h; and desolvation gas flow, 800 L/h. Unit resolution was applied to each quadrupole. For qualitative studies, the acquisition was performed in full scan and SIR (single ion recording), while for cycloclavine **6** quantification a MRM (multiple reaction monitoring) method was used. Based on collision induce dissociation studies the first quadrupole was set on the parent ion $[M + H]^+$ = 239.2 whilst the third quadrupole was set to *m/z* 144.1 (cone voltage 36 V; collision energy 24 V).

High resolution mass spectra were acquired on a Synapt G2 HDMS mass spectrometer (Waters) operated in positive mode with a scan time of 1.5 s in the mass range of 100-600 m/z. Samples (5-7 µL) were injected onto an Acquity BEH C18 reversed phase column (1.7 µm, 2.1x50mm, Waters) and eluted with a gradient of 0-90% acetonitrile in water/0.1% formic acid over a time of 11 min at a flow rate of 0.4 mL/min. Capillary voltage was 0.5 V, cone voltage 30 V, source temperature 100°C, desolvation temperature 250°C. Leu-enkephalin peptide was used to generate a dual lock-mass calibration with $[M + H]^+$ = 556.2766 and *m/z*= 278.1135 measured every 10 s during the run.

SI-1.12 Nuclear magnetic resonance (NMR) spectroscopy

NMR experiments of cycloclavine **6** and festuclavine **4** were performed respectively in $CDCl_3$ and DMSO-d_6 at 25°C using a Bruker Avance III 600MHz NMR spectrometer

equipped with a 1.7mm cryogenic TCI probe. The structure of cycloclavine **6** was solved by means ^{1}H-NMR, ^{13}C-NMR, ^{1}H,^{1}H-ROESY, and ^{1}H,^{13}C-HMBC.

SI-2 Supporting Figures

Figure S1 Organization of the *Aspergillus japonicus* cycloclavine 6 gene cluster

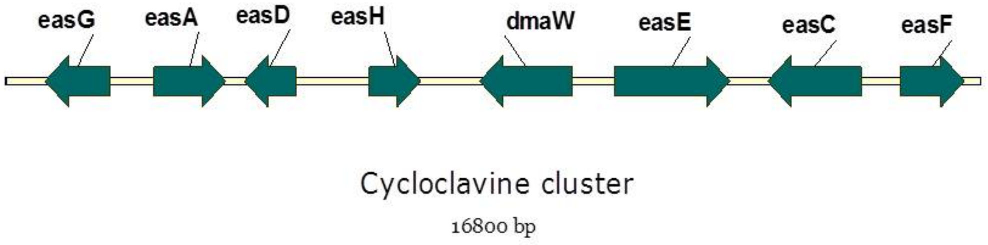

Figure S2 Temperature dependent production of chanoclavine-I 2
A yeast strain expressing the biosynthetic pathway to chanoclavine-I **2** (Nielsen et al. 2014)[5] was grown for 72 hours at different temperatures to evaluate the effect on *N*-Me-DMAT **1** and chanoclavine-I production. Lower temperatures resulted in lower biomass production and lower final *N*-Me-DMAT **1** titer. In contrast, the final titer of chanoclavine-I **2** increased at lower temperatures, likely reflecting an improved activity of the enzymes (EasC/EasE) involved in *N*-Me-DMAT **1** to chanoclavine-I **2** conversion.

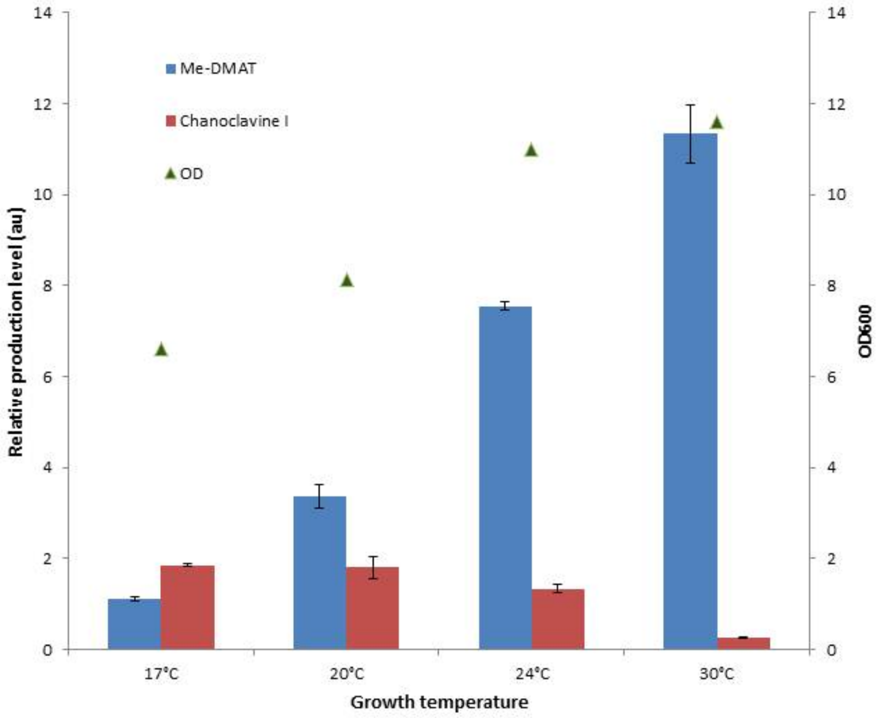

Figure S3 A chanoclavine-I 2 producing yeast was used for ectopic expression of combinations of ergot alkaloid genes

Expression of *easD* and *easG* from *Aspergillus japonicus* (i) led to production of chanoclavine-I aldehyde **3** ($[M + H]^+$ = 255), as would be expected from the activity of *easD*. However, the forward reaction was not complete, as seen by the concommittant accumulation of chanoclavine-I **2** ($[M + H]^+$ = 257). No new products were detected, and none of the two products seem to be a substrate for *easG*. Expression of *easD* in combination with *easA* (ii), both from *Aspergillus japonicus*, resulted in production of a new compound, with an $[M + H]^+$ of 257. This compound was not purified, but is likely to be dihydro-chanoclavine-I aldehyde **7**, which was also seen in the *in vitro* assay (Figure S21). Expression of *easD* (*Aspergillus japonicus*) in combination with *easA* and *easG* from either *Aspergillus japonicus* (iii) or from *Aspergillus fumigatus* (iv) resulted in the production of festuclavine **4** ($[M + H]^+$ = 241).

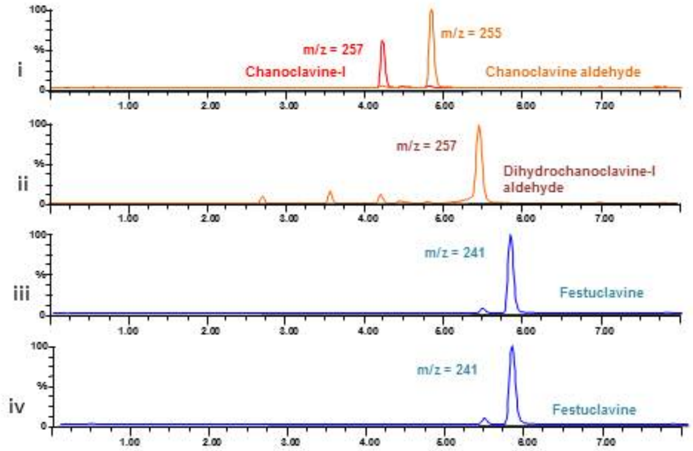

Figure S4 The ratio between cycloclavine 6 and festuclavine 4 production depends on the copy number of *easH*

A yeast strain expressing the entire cycloclavine pathway, comprising a single copy of *easH* (eash x 1), produced cycloclavine **6** and festuclavine **4** approximately in the ratio 3:2. Addition of 1, 2, or 3 extra gene copies increased this ratio in a dose dependent manner. With 3 additional copies (easH x 4) the ratio was more than 7:1 of cycloclavine **6** to festuclavine **4.**

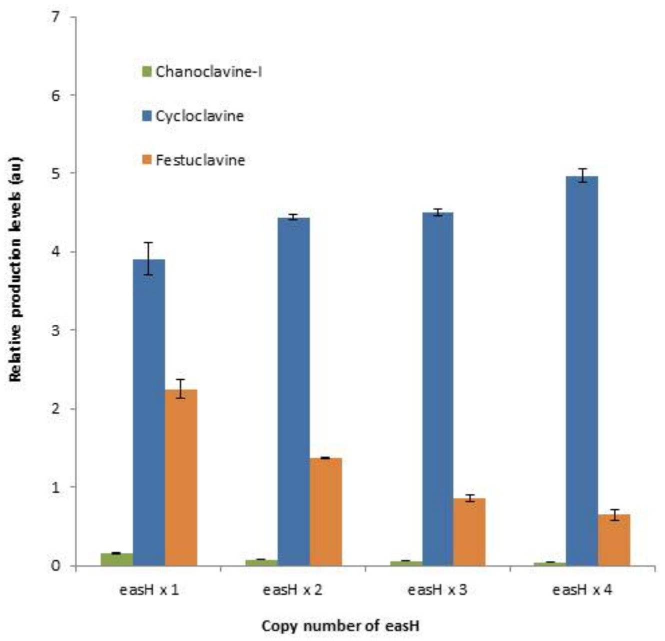

Figure S5 NMR data (^{1}H- and ^{13}C-NMR chemical shifts)

Cycloclavine **6**: ^{1}H NMR (600 MHz, $CDCl_3$) δ 9.42 (br s, 1H), 7.25 (br d, *J*=8.75 Hz, 1H), 7.11 (t, *J*=7.62 Hz, 1H), 6.95 (br s, 1H), 6.82 (d, *J*=7.20 Hz, 1H), 4.00-4.07 (m, 1H), 3.69 (br d, *J*=11.67 Hz, 1H), 3.32 (br d, J=13.69 Hz, 1H), 3.21 (br s, 1H), 3.08 (br d, *J*=10.40 Hz, 1H), 2.89 (br s, 3H), 1.87 (br d, *J*=5.84 Hz, 1H), 1.78 (s, 3H), 0.76 (br d, *J*=6.07 Hz, 1H); ^{13}C NMR (150 MHz, $CDCl_3$) δ 119.5 (1C), 133.5 (1C), 130.4 (1C), 127.9 (1C), 122.7 (1C), 110.2 (1C), 109.5 (1C), 108.5 (1C), 70.5 (1C), 63.0 (1C), 39.0 (1C), 33.1 (1C), 26.2 (1C), 22.6 (1C), 22.4 (1C), 15.2 (1C). Festuclavine **4**, ^{1}H NMR (600 MHz, DMSO-d_6) δ 10.98 (br s, 1H), 7.24 (d, *J*=8.07 Hz, 1H), 7.10 (br s, 1H), 7.09 (t, 1H), 6.85 (d, *J*=6.97 Hz, 1H), 3.48 (br d, *J*=11.00 Hz, 2H), 3.45-3.51 (br m, 1H), 3.26 (br t, *J*=9.90 Hz, 1H), 3.16 (br s, 1H), 2.93-2.95 (m, 1H), 2.94 (s, 3H), 2.76 (br t, *J*=11.00 Hz, 1H), 2.65 (br d, *J*=12.84 Hz, 1H), 2.16-2.21 (m, 1H), 1.14-1.22 (m, 1H), 1.01 (d, *J*=6.60 Hz, 3H); ^{13}C NMR (150 MHz, DMSO-d_6) δ 133.5 (1C), 129.9 (1C), 125.5 (1C), 122.6 (1C), 119.6 (1C), 112.9 (1C), 109.8 (1C), 107.4 (1C), 65.9 (1C), 61.3 (1C), 40.8 (1C), 38.6 (1C), 34.2 (1C), 28.5 (1C), 24.1 (1C), 18.5 (1C).

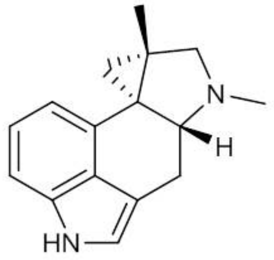

Cycloclavine
6

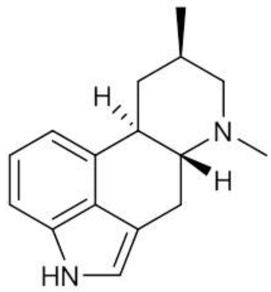

Festuclavine
4

Figure S6 Key HMBC correlations of cycloclavine 6

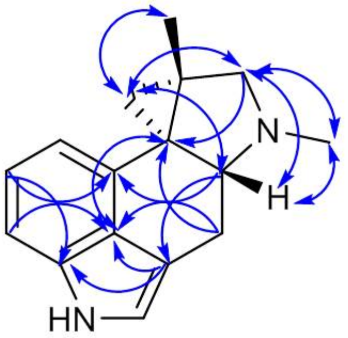

6

Figure S7 Key ROESY correlations of cycloclavine 6

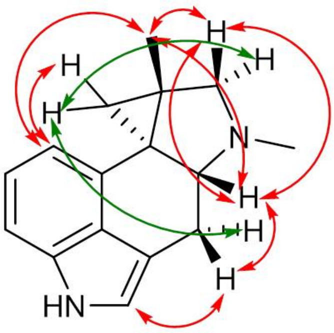

6

Figure S8 Alignment of EasH (*A. japonicus*) and homologs

(1) Phytanoyl-CoA 2-hydroxylase (PAHX); (2) *A. japonicus* EasH (EasH_Aj); (3) *Claviceps purpurea* EasH (EasH_Cp); (4) WelO5. Active site residues are highlighted in red boxes. Alignment was performed using MUSCLE v3.8.31 software (Edgar, 2004).[9]

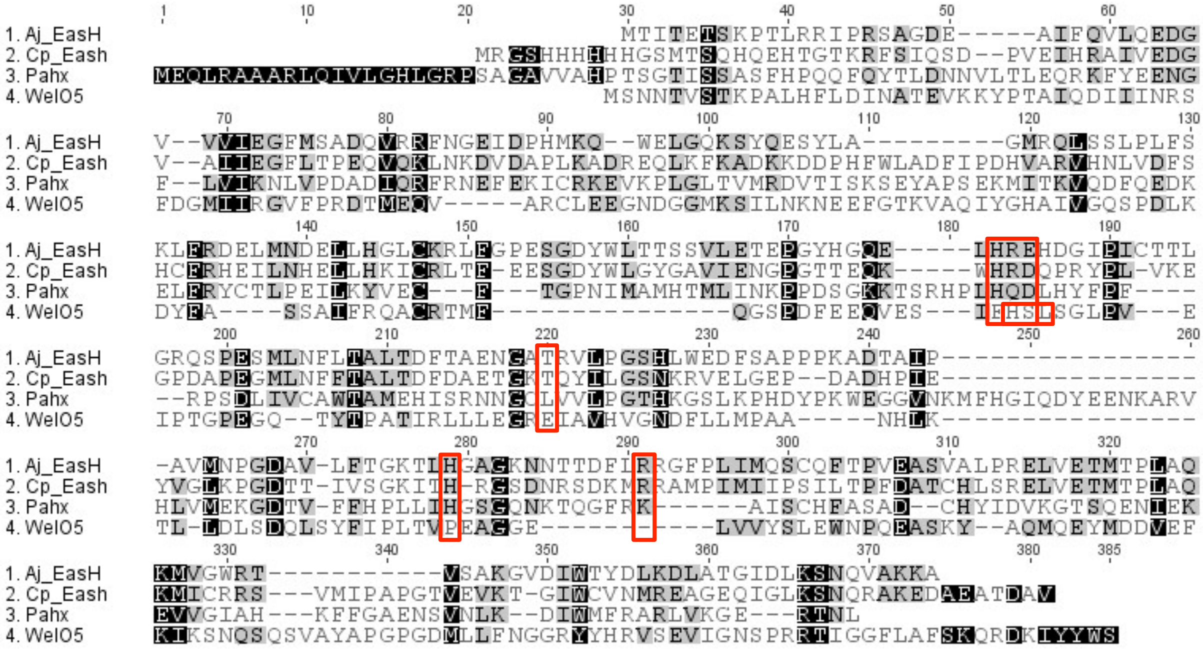

Figure S9 SDS-PAGE of heterologously expressed proteins

(a) EasA (*A. fumigatus*) (45 kDa); (b) EasG (*A. fumigatus*) (32 kDa); (c) EasA (*A. japonicus*) (45 kDa); (d) EasG (*A. japonicus*) (33 kDa); (e) EasH (*A. japonicus*) (58 kDa); (*) Run Blue Prestained markers (Expedeon).

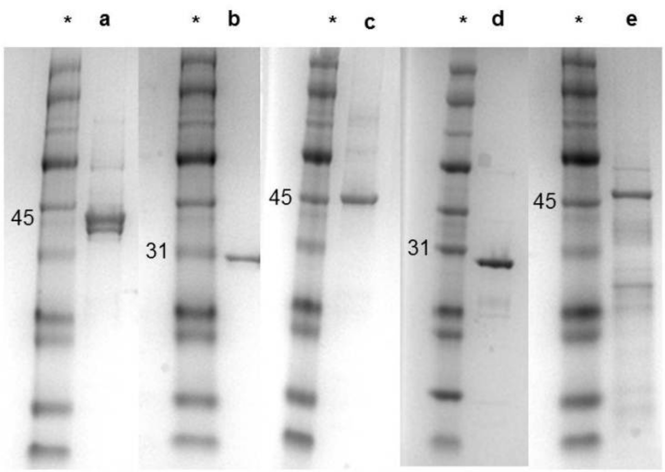

Figure S10 *In vitro* assays with EasA, EasH, EasG and varying co-factor combinations. In all cases the substrate is chanoclavine-I aldehyde **3**. Conditions are as described in the methods for *in vitro* endpoint assays unless otherwise specified. (a) 500 μM $NADP^+$, 100 μM L-ascorbic acid, 100 μM α-ketoglutaric acid (without 10μM $FeSO_4$*7H_2O) = no cycloclavine **6**, monitoring at $[M+H]^+$ 239; (b) 500 μM $NADP^+$, 100 μM L-ascorbic acid, 100 μM α-ketoglutaric acid (without 10μM $FeSO_4$*7H_2O) = festuclavine **4**, monitoring at $[M+H]^+$ 241; (c) 500 μM $NADP^+$, 10 μM $FeSO_4$*7H_2O, 100 μM α -ketoglutaric acid (without 100μM L-ascorbic acid) = cycloclavine **6**; (d) 500 μM $NADP^+$, 100 μM L-ascorbic acid, 10 μM $FeSO_4$*7H_2O (without 100 μM α -ketoglutaric acid) = no cycloclavine **6**, monitoring at $[M+H]^+$ 239; (e) 500 μM $NADP^+$, 100 μM L-ascorbic acid, 10 μM $FeSO_4$*7H_2O (without 100 μM α -ketoglutaric acid) = festuclavine **4**, monitoring at $[M+H]^+$ 241; (f) 100 μM L-ascorbic acid, 10 μM $FeSO_4$*7H_2O, 100 μM α -ketoglutaric acid (without 500 μM $NADP^+$) = chanoclavine-I aldehyde **3** (starting material); (g) 500 μM NADP+, 100 μM L-ascorbic acid, 10 μM $FeSO_4$*7H_2O, 100 μM α -ketoglutaric acid + 500 μM NADPH (additional) = festuclavine **4** (an excess of NADPH results in more reductive product than cycloclavine **6** product).

EasH requires all of the co-factors (excluding L-ascorbic acid which is only preventing from iron oxidation) to produce cycloclavine **6**. This fact supports hypothesis that EasH is a Fe(II) and α -ketoglutarate dependent hydroxylase.

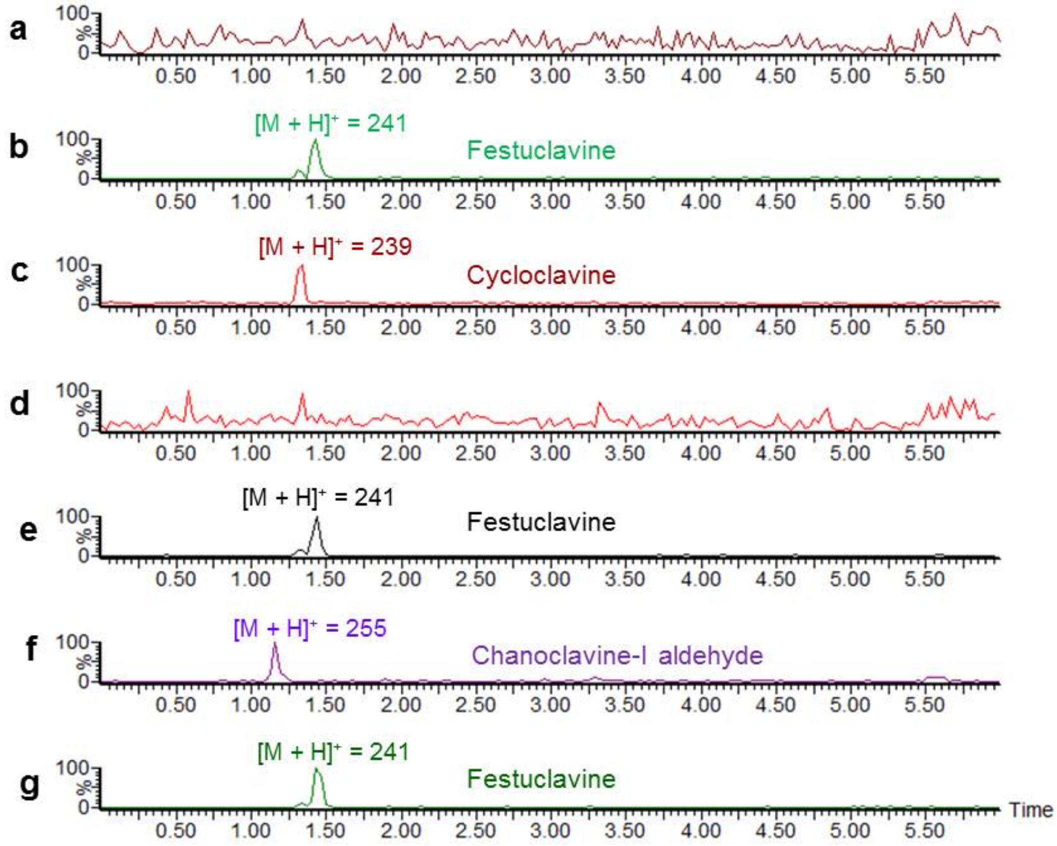

Figure S11 Standard curve for cycloclavine 6.
Standard curve for cycloclavine **6** concentration and peak area response observed by LC-MS. The concentrations 1.25-0.039 µM represent the full range of cycloclavine **6** product that was analyzed by mass spectrometry.

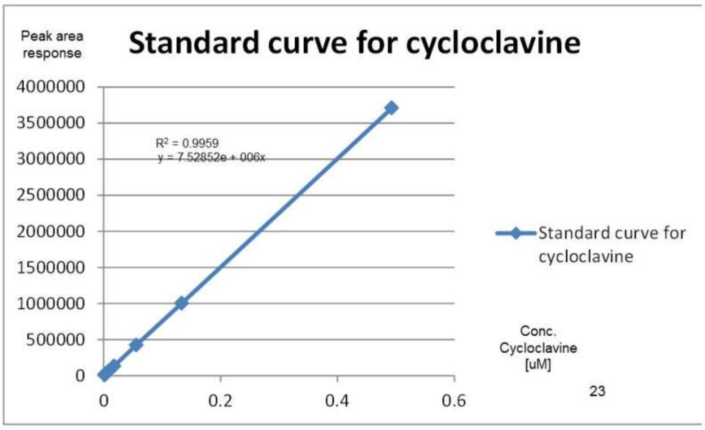

Figure S12 Time course of EasH assays
Conditions are as described in the methods for *in vitro* endpoint assays unless otherwise specified. Cycloclavine **6** concentration increases with time.

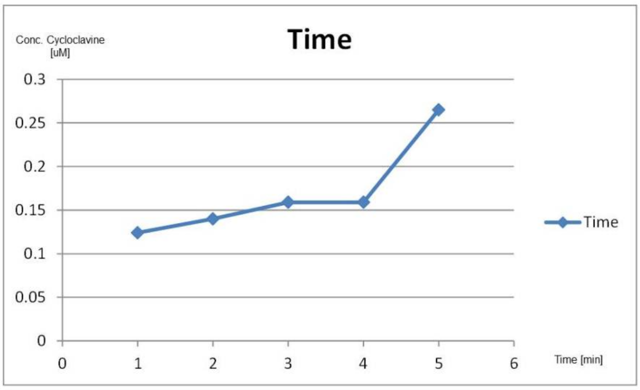

Figure S13 Concentration of cycloclavine 6 increases with increasing EasH concentration and with time

Conditions are as described in the methods for *in vitro* endpoint assays unless otherwise specified.

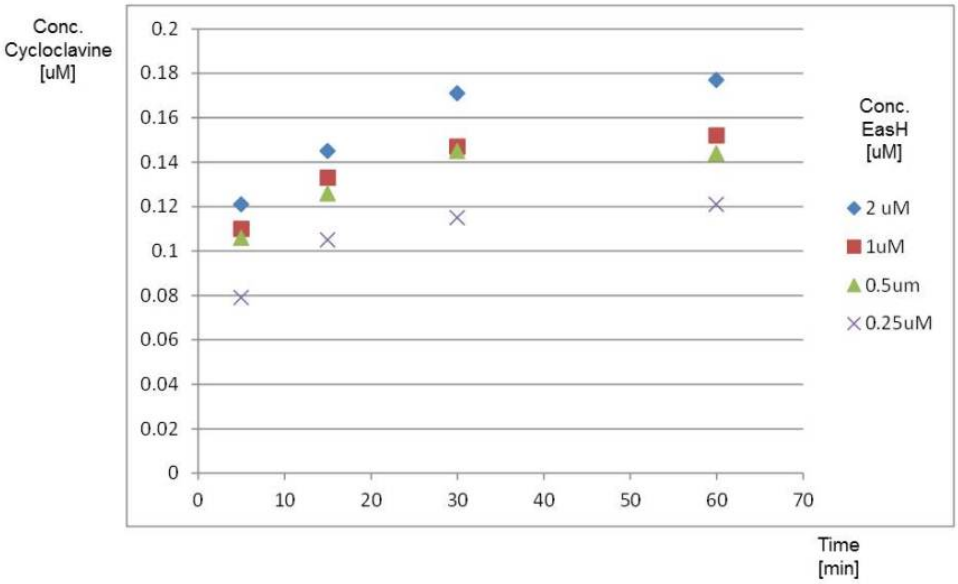

Figure S14 Cycloclavine 6 formation dependence on concentration of chanoclavine-I aldehyde 3 and time

Concentration of cycloclavine **6** increases with increasing chanoclavine-I aldehyde **3** concentration and with time. Conditions are as described in the methods for *in vitro* endpoint assays unless otherwise specified.

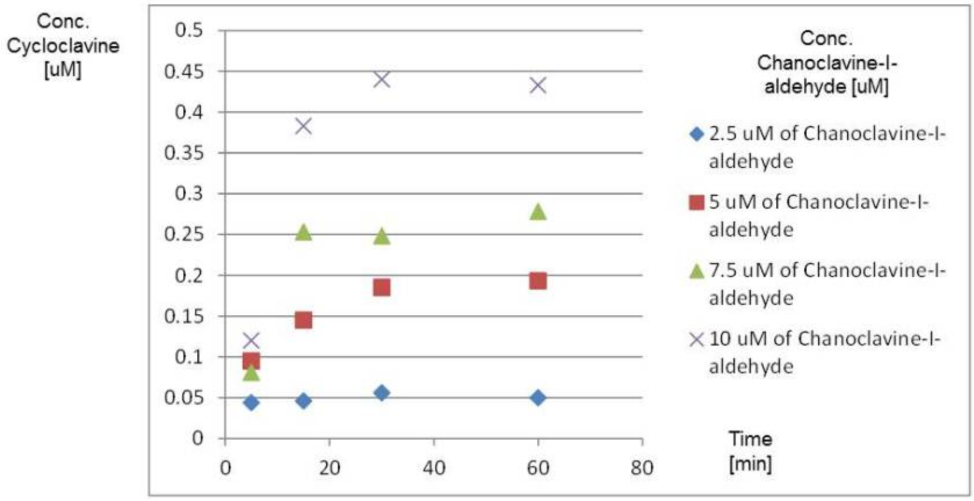

Figure S15 The pH optimum of cycloclavine 6 formation by EasA, EasG and EasH (*A. japonicus*) was determined to be pH = 7
Assay conditions are as described in the methods for *in vitro* endpoint assays. Buffers used were: 100 mM K_2HPO_4 buffers at pH: 5, 6, 7 and 8 were used.

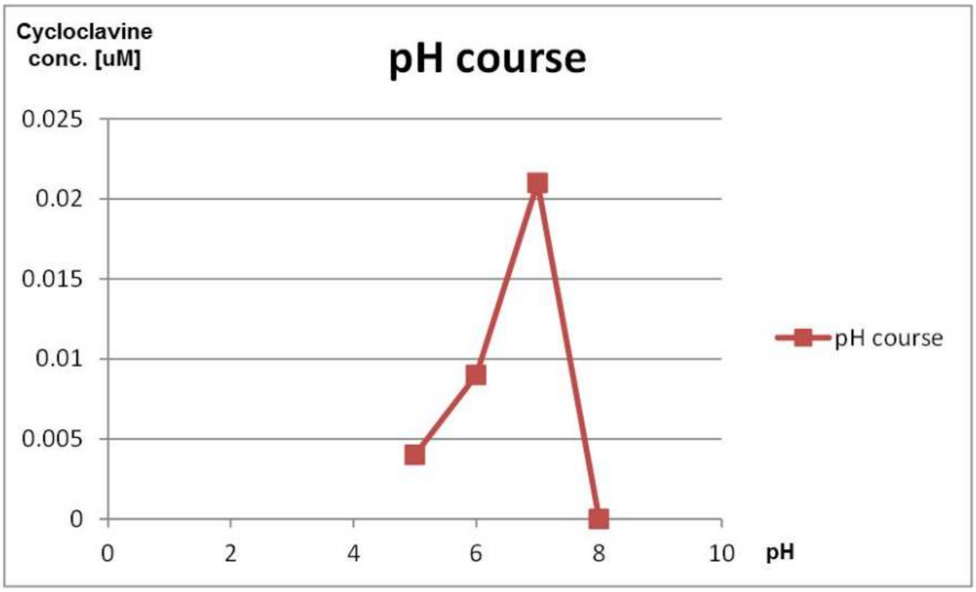

Figure S16 Assays with chanoclavine-I aldehyde 3 and different enzyme combinations
Conditions are as described in the methods for *in vitro* endpoint assays unless otherwise specified. (a) EasA (*A. japonicus*) + EasH (*A. japonicus*) = starting material consumption, but no festuclavine **4** or cycloclavine **6**; (b) EasG (*A. japonicus*) + EasH (*A. japonicus*) = starting material consumption, but no festuclavine **4** $[M + H]^+ = 241$ or cycloclavine **6** $[M + H]^+ = 239$; (c) EasA (*A. japonicus*) + EasG (*A. japonicus*) + NADPH = festuclavine **4**; (d) EasA (*A. japonicus*) + EasG (*A. japonicus*) = chanoclavine-I aldehyde **3** (starting material).
Cycloclavine **6** synthesis is possible only in the presence of all of the enzymes – Aj_EasA, Aj_EasH and Aj_EasG. Festuclavine **4** production requires only Aj_EasA and Aj_EasG.

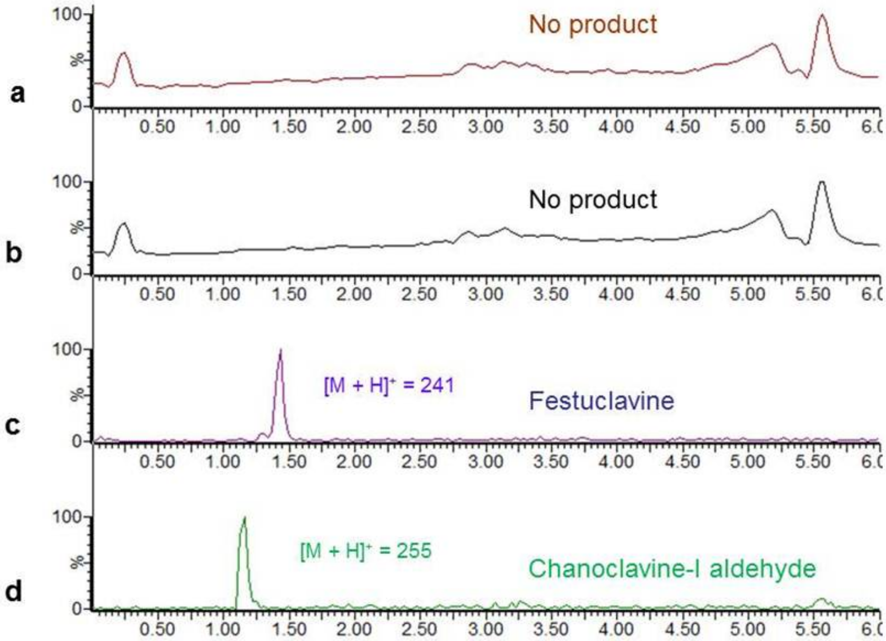

Figure S17 Cycloclavine 6 and festuclavine 4 production with different ratio of enzymes

Cycloclavine **6** and festuclavine **4** production with different ratio of enzymes: EasA: EasG: EasH (*A. japonicus*). The highest concentration of cycloclavine **6** was observed with ten-fold excess of EasH. The highest concentration of festuclavine **4** was observed when Aj_EasA was in 10-fold excess.

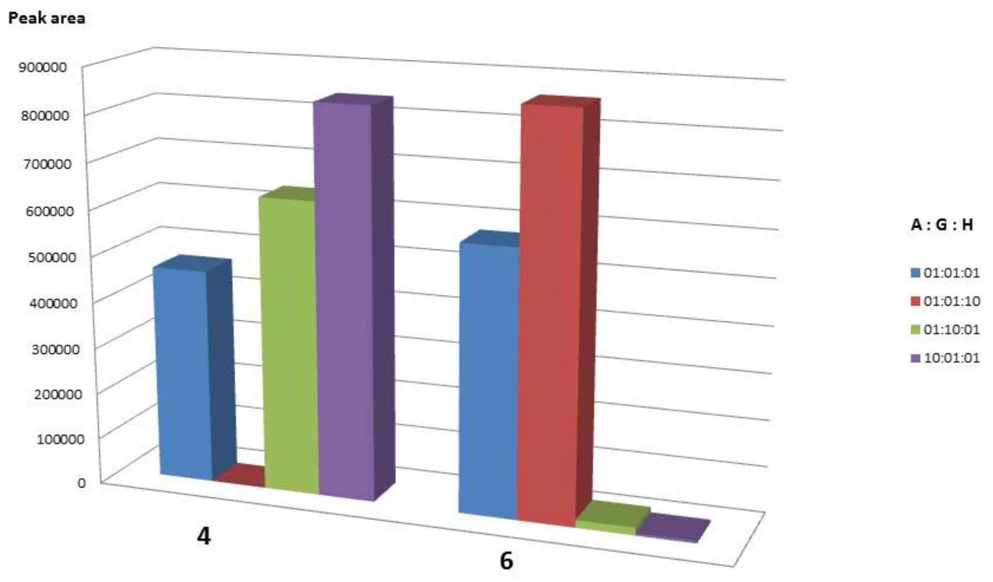

Figure S18 UV spectra of EasH

(a) EasH which contains bound $NADP^+$ (after first purification by FLAG-tag); (b) EasH subjected to a second purification step (by gel filtration column), which loses activity in within 48 hours, appears to have lost the $NADP^+$ cofactor during the second purification step.

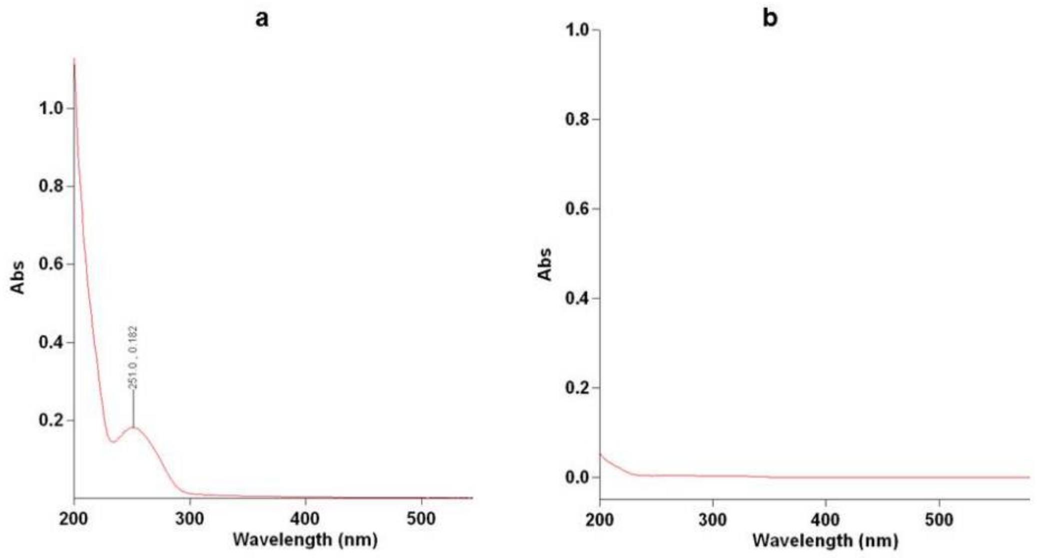

Figure S19 Assays with EasH (no EasA or EasG)
(a) Chanoclavine-I aldehyde **3**, $[M + H]^+$ = 255 (b) Agroclavine **5**, $[M + H]^+$ = 239; (c) Festuclavine **4**, $[M + H]^+$ = 241. Only the starting materials are left after 2 hours of reaction. Conditions are as described in the methods for *in vitro* endpoint assays unless otherwise specified.
Chanoclavine-I aldehyde **3**, agroclavine **5** or festuclavine **4** are clearly not the substrates for cycloclavine **6** production by Aj_EasH (alone).

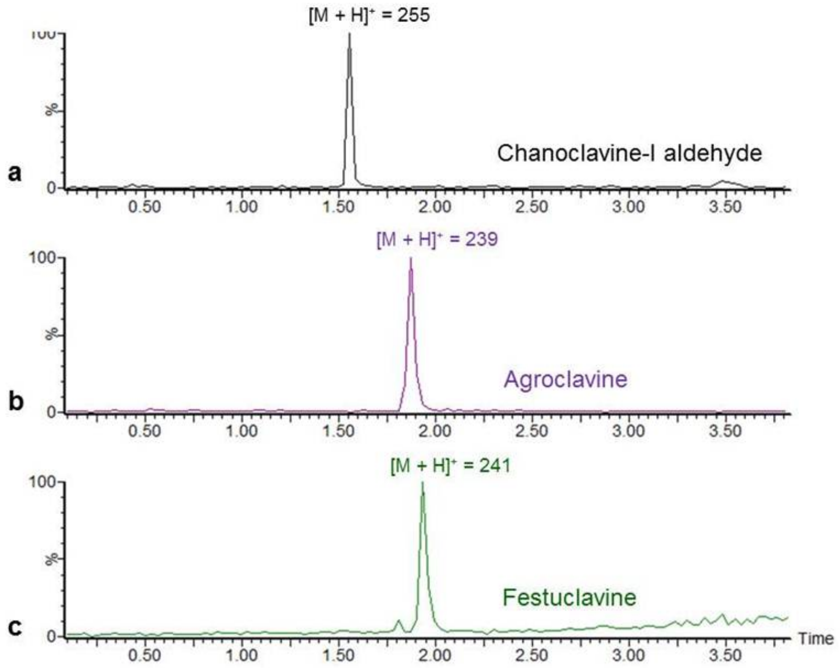

Figure S20 Detection of intermediate in *in vitro* cycloclavine biosynthetic reactions
Conditions are as described in the methods for *in vitro* endpoint assays unless otherwise specified. (a) EasA (*A. japonicus*) + EasH (*A. japonicus*) + co-factors = intermediate **10** (M^+= 237); (b, c) EasA (*A. japonicus*) + EasH (*A. japonicus*) + (after 2 h) EasG (*A. japonicus*) yielded cycloclavine **6** + festuclavine **4.**

Upon incubation of chanoclavine-I aldehyde **3** with Aj_EasA and Aj_EasH (with co-factors), intermediate with M^+ = 237 has been observed. Addition of Aj_EasG generates cycloclavine **6** which supports mechanism proposed in figure 3 (main text).

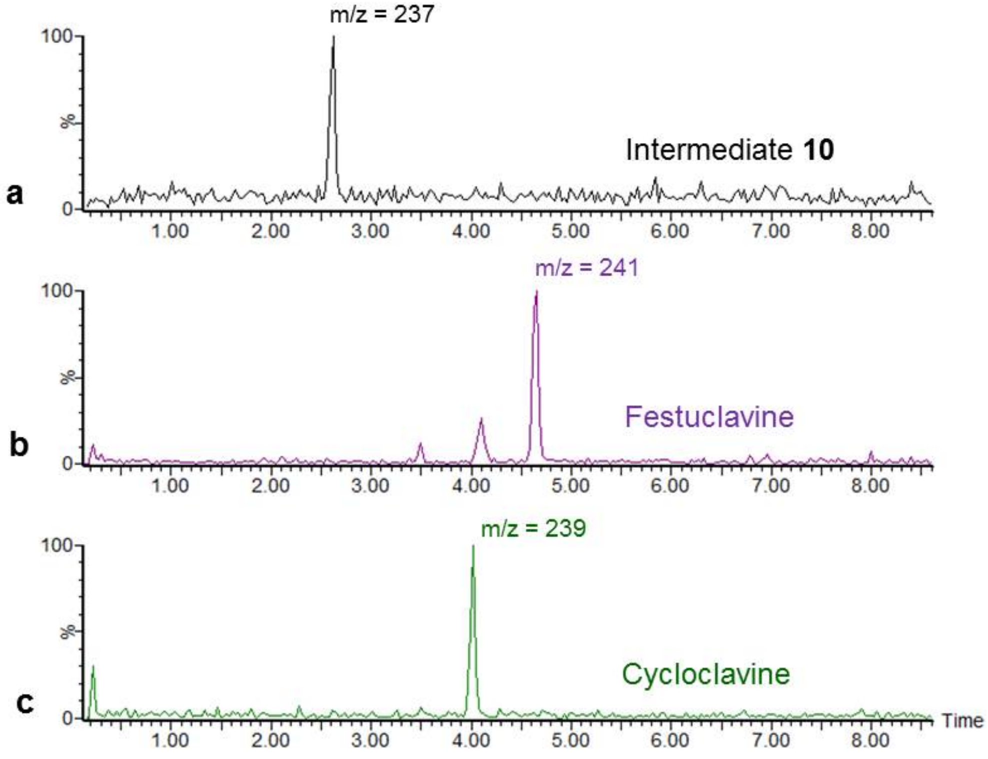

Figure S21 Intermediates observed in assays with enzymes cloned from both *A. japonicus* and *A. fumigatus*

Conditions are as described in the methods for *in vitro* endpoint assays unless otherwise specified. (a) EasA (*A. fumigatus*) (5 fold excess) + EasG (*A. japonicus*) + EasH (*A. japonicus*) + co-factors = cycloclavine **6** $[M + H]^+ = 239$ + iminium intermediate **8** $M^+ = 239$; (b) EasA (*A. fumigatus*) + EasH (*A. japonicus*) + co-factors = dihydrochanoclavine-I aldehyde **7** $[M + H]^+ = 257$; (c, d) EasA (*A. fumigatus*) + EasG (*A. japonicus*)+ EasH (*A. japonicus*) + co-factors = cycloclavine **6** $[M + H]^+ = 239$ + festuclavine **4** $[M + H]^+ = 241$; (e, f) EasA (*A. japonicus*) + EasG (*A. fumigatus*) + EasH (*A. japonicus*) = cycloclavine **6** $[M + H]^+ = 239$ + festuclavine **4** $[M + H]^+ = 241$.

We observed iminium species **8** and dihydrochanoclavine-I aldehyde **7** intermediate which suggests that EasA from *A. fumigatus* effects on chanoclavine-I aldehyde **3** in a similar way as EasA from *A. japonicus* (a, b). Moreover, mixing EasA and EasG from two different species (*A. japonicus* and *A. fumigatus*) in the presence of EasH (*A. japonicus*) didn't change the result of the assays – in both cases cycloclavine **6** and festuclavine **4** (by-product) were synthesised (c-f).

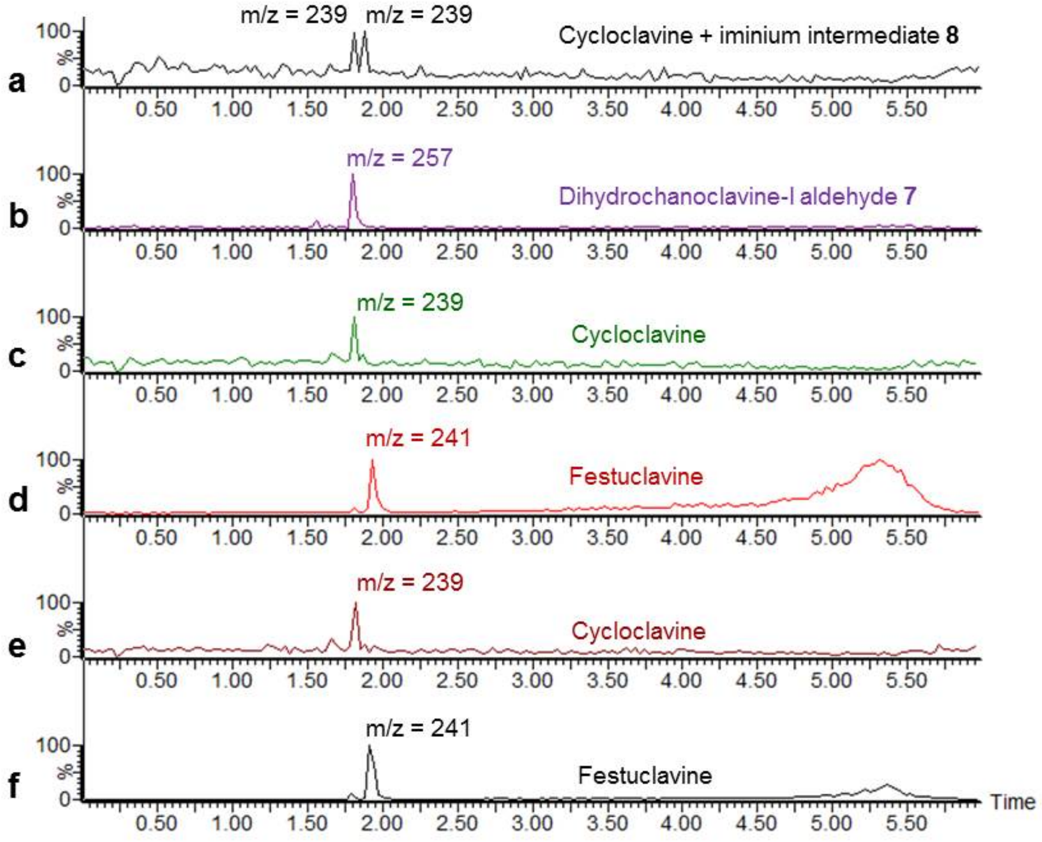

Figure S22 "Trapping" of iminium intermediate **8** by selective reduction reaction with $NaBD_3CN$ yielded deuterium labelled festuclavine **11.** LC-MS chromatograms of: (a) Deuterated festuclavine **11.** (b) Festuclavine **4** standard.

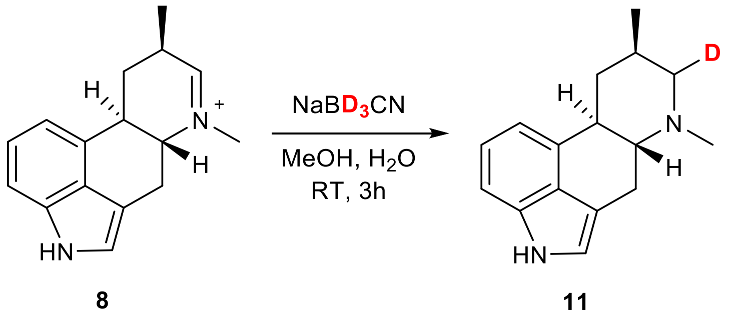

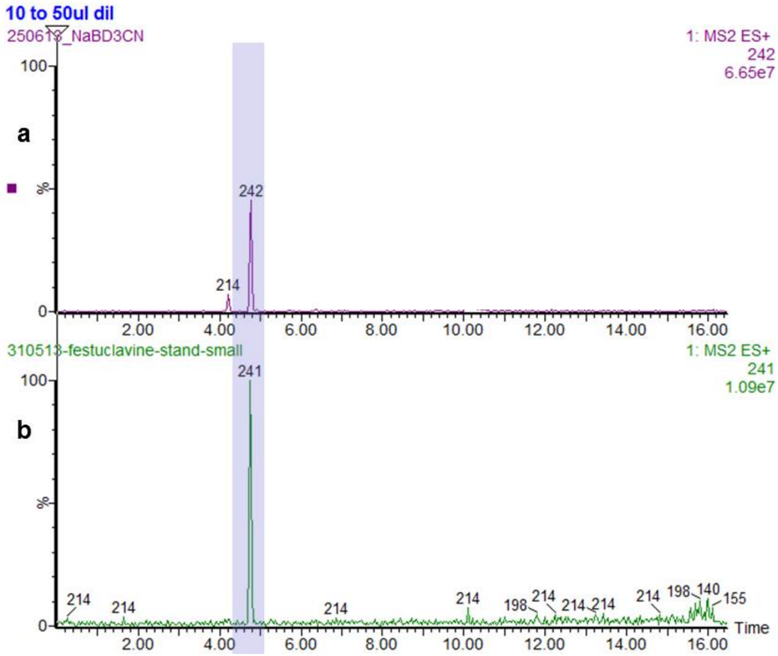

SI-3 Supporting Tables

Table S1. Integration constructs inserted into *S. cerevisiae* S288c chromosomal DNA

Chromosome	Construct
Chr. VIII	YHRCΔ14::TP-ScPdi1/GC-ScFad1/TE-AjDmaW/PF-AjEasC/PA-AjEasD/Ble
Chr. XIII	YMRWΔ15::GC-AjEasH/PA-AjDmaW/PF-AjEasE/TE-Af-EasF/TP-AjEasC/HphMX
Chr. XIV	YNRCΔ9::GC-AjEasH/PA-AjEasC/TE-AjEasG/TP-AjEasD/NatMX
Chr. XV	YORWΔ22::GC-AjEasH/PA-AjEasD/PF-AjEasC/KlUra3
Chr. XVI	YPRCτ3::GC-AjEasG/PA-AjEasA/PF-AjEasH/TE-AjDmaWC/NatMX

Table S2. ^{1}H-NMR and ^{13}C-NMR shifts of cycloclavine 6

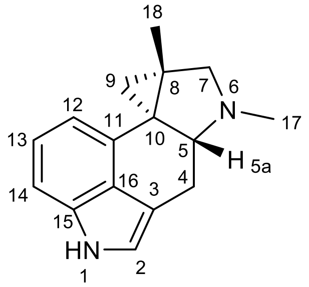

6

**Atom No**	**^{1}H-NMR (δ (^{1}H) [ppm], Multiplicity, No of protons)**	**^{13}C-NMR (δ (^{13}C) [ppm])**
1	9.42 (br s, 1H)	-
2	6.95 (s, 1H)	119.5
3	-	108.5
4	3.21 (br t, 1H) / 3.32 (br d, *J*=13.69 Hz, 1H)	22.4
5	-	70.5
5a	3.69 (br d, *J*=11.67 Hz, 1H)	-
6	-	-
7	3.08 (br d, *J*=10.40 Hz, 1H) / 4.05 (br t, 1H)	63.0
8	-	26.2
9	0.76 (d, *J*=6.07 Hz, 1H) / 1.87 (d, *J*=6.07 Hz, 1H)	22.6
10	-	33.1
11	-	130.4
12	6.82 (d, *J*=7.20 Hz, 1H)	110.2
13	7.11 (br t, *J*=7.62 Hz, 1H)	122.7
14	7.25 (br d, *J*=8.75 Hz, 1H)	109.5
15	-	133.5
16	-	127.9
17	2.89 (s, 3H)	39.0
18	1.78 (s, 3H)	15.2

Table S3. Exact Masses of compounds were determined by electrospray (ESI) (operated in positive mode) high resolution mass spectrometry (MS)

**Compound**	**Theoretical mass $[M + H]^+$ [Da]**	**Observed mass $[M + H]^+$ [Da]**	**Deviation of Theoretical from Observed Mass (ppm)**	**Molecular Formula**
Chanoclavine-I aldehyde **3**	255.1497	255.1498	0.39	$C_{16}H_{18}N_2O$
Festuclavine **4**	241.1704	241.1712	3.32	$C_{16}H_{20}N_2$
Cycloclavine **6**	239.1548	239.1541	2.93	$C_{16}H_{18}N_2$

Table S4. Operating conditions for the fed-batch fermentation

**Parameter**	**Set-point**
Volume of liquid [L]	0.32 – 0.39
Temperature [° C]	20
pH	5.85
Agitation speed [rpm]	650
Air flow rate [vvm]	1

Table S5. Composition of the "SC" medium for fermentation

	Concentration [g/L]
Total carbon source	20
Glucose x H_2O	22
YNB* with ammonium, without amino acids	6.7
Amino Acid drop out mix (KAISER) (-His -Leu -Ura)	1.47
L-Histidine	0.076
L-Leucine	0.38
L-Methionine	0.076
Vitamin stock solution (see Table S6)	1 [ml/L]

*Yeast Nitrogen Base

Table S6. Composition of feed solution, using NH_4OH as nitrogen source

	Concentration [g/L]
Total carbon source	600
Glucose x H_2O	660
YNB* without ammonium, without amino acids	10.2
Amino Acid drop out mix (KAISER) (-His -Leu -Ura)	4.41
L-Histidine	0.228
L-Leucine	1.14
L-Methionine	0.228
KH_2PO_4	10.8
$MgSO_4$ x $7H_2O$	6.12
K_2SO_4	4.2
Na_2SO_4	0.336
Vitamin stock solution (see Table S6)	30 [ml/L]
Trace metal stock solution (see Table S7)	15 [ml/L]

*Yeast Nitrogen Base

Table S7. Composition of the stock trace metal solution

	Concentration [g/L]
Na_2EDTA x $2H_2O$	15
$ZnSO_4$ x $7H_2O$	4.5
$FeSO_4$ x $7H_2O$	3
$CaCl_2$ x $2H_2O$	3
$MnCl_2$ x $4H_2O$	1
$CoCl_2$ x $6H_2O$	0.32
$CuSO_4$ x $5H_2O$	0.3
Na_2MoO_4 x $2H_2O$	0.4
H_3BO_3	1
KI	0.1

Table S8. Composition of the stock vitamin solution

	Concentration [g/L]
d-Biotin	0.1
Ca-Pantothenate	2
Thiamine-HCl	2
Pyridoxine-HCl	2
Nicotinic acid	2
p-Aminobenzoic acid	2
m-Inositol	0.05

References for Supporting Information

1 Cheng, J. Z., Coyle, C. M., Panaccione, D. G. & O'Connor, S. E. A role for Old Yellow Enzyme in ergot alkaloid biosynthesis. *J. Am. Chem. Soc.* **132**, 1776-1777 (2010).

2 Petersen, T., Brunak, S., von Heijne, G. & Nielsen, H. SignalP 4.0: discriminating signal peptides from transmembrane regions. *Nat. Methods* **8**, 785-786 (2011).

3 Schröder, H. & Hoff, B. Gene Cluster for Biosynthesis of Cycloclavine. *PCT Int. Appl. WO 2012116935 A2 20120907* (2012).

4 Sikorski, R. S. & Hieter, P. A. System of shuttle vectors and yeast host strains designed for efficient manipulation of DNA in *Saccharomyces cerevisiae*. *Genetics* **122**, 19-27 (1989).

5 Nielsen, C. A. F., Folly, C., Hatsch, A., Molt A., Schröder, H., O'Connor, S. E. & Naesby, M. The important ergot alkaloid intermediate chanoclavine-I produced in the yeast. *Microbial Cell Fact.* **13**, 1-11 (2014).

6 Flagfeldt, D. B., Huang, L. & Nielsen, J. Characterization of chromosomal integration sites for heterologous gene expression in *Saccharomyces cerevisiae*. *Yeast* **26**, 545–551 (2009).

7 Cubillos, F. A., Louis, E. J. & Liti, G. Generation of a large set of genetically tractable haploid and diploid Saccharomyces strains. *FEMS Yeast Res.* **9**, 1217-1225 (2009).

8 Cheng, J. Z., Coyle, C. M., Panaccione, D. G. & O'Connor, S. E. Controlling a structural branch point in ergot alkaloid biosynthesis. *J. Am. Chem. Soc.* **132**, 12835-12837 (2010).

9 Edgar, R. C. MUSCLE: multiple sequence alignment with high accuracy and high throughput. *Nucleic Acids Res.* **32**, 1792-1797 (2004).
